# Documentation of Driving Status and of Fitness to Drive Following Admission of Patients to Clock View Hospital - How Are We Doing?

**DOI:** 10.1192/bjo.2022.305

**Published:** 2022-06-20

**Authors:** Azmeralda Abraheem, Enrica Barnes, Ryan Hendry, Cameron Martin, Declan Hyland

**Affiliations:** Mersey Care NHS Foundation Trust, Liverpool, United Kingdom

## Abstract

**Aims:**

Developing a mental illness and being commenced on psychotropic medication are factors that may interfere with the ability of an individual to drive safely as both can impact information processing, psychomotor actions and social interpretation. The Driver and Vehicle Licensing Agency (DVLA) suggests that certain medical conditions require driving licence holders to notify them for further assessment of their ability to drive. DVLA notifiable mental disorders include psychosis, schizophrenia, bipolar disorder, dementia and personality disorders. The doctor's legal duty is to assess the patient for any relevant diagnosis, inform the patient of their duty to report their medical condition to the DVLA and for the doctor to comply with the legal duty to inform the DVLA of any patient who won't or can't notify the DVLA of their medical condition. The authors conducted a quality improvement project to evaluate and improve the number of fitness to drive assessments completed for patients admitted to the five wards (three general adult, one older adult and the Psychiatric Intensive Care Unit) at Clock View Hospital.

**Methods:**

The electronic (RiO) record for each inpatient on the five wards was scrutinised for: whether the patient's driving status was established on admission; whether the patient was notified of the DVLA rules if they did drive; whether the patient agreed to fulfil their duty of notification and, in instances where they were not, whether the medical professional had taken appropriate steps to address this.

**Results:**

74 patients on the five wards were included in the sample. Only nine of the 74 patients had driving status documented on admission. Three of these nine patients were noted to be driving or learning to drive and were not notified of the DVLA rules. Four of the nine patients were no longer driving and so discussion about DVLA guidance was unnecessary. The remaining two patients were confirmed to be driving and informed of the DVLA regulations. Both patients agreed to comply and therefore no further action was indicated.

**Conclusion:**

A review of current practice indicates a deficit in incorporating driving status and fitness to drive assessment into the clerking proforma following admission to Clock View Hospital. The second half of this cycle will implement change and raise awareness amongst inpatient medical and nursing staff of the need to consider this important issue prior to discharge. A re-assessment of the effectiveness of these changes will be carried out in the future.

